# Bio‐field array: a dielectrophoretic electromagnetic toroidal excitation to restore and maintain the golden ratio in human erythrocytes

**DOI:** 10.14814/phy2.13722

**Published:** 2018-06-10

**Authors:** Marcy C. Purnell, Matthew B.A. Butawan, Risa D. Ramsey

**Affiliations:** ^1^ The Loewenberg College of Nursing University of Memphis Memphis Tennessee; ^2^ School of Health Studies University of Memphis Memphis Tennessee

**Keywords:** dielectrophoretic field flow fractionation, erythrocyte, Golden Ratio, rheological alterations, toroid

## Abstract

Erythrocytes must maintain a biconcave discoid shape in order to efficiently deliver oxygen (O_2_) molecules and to recycle carbon dioxide (CO_2_) molecules. The erythrocyte is a small toroidal dielectrophoretic (DEP) electromagnetic field (EMF) driven cell that maintains its zeta potential (*ζ*) with a dielectric constant (*ԑ*) between a negatively charged plasma membrane surface and the positively charged adjacent Stern layer. Here, we propose that zeta potential is also driven by both ferroelectric influences (chloride ion) and ferromagnetic influences (serum iron driven). The Golden Ratio, a function of Phi φ, offers a geometrical mathematical measure within the distinct and desired curvature of the red blood cell that is governed by this zeta potential and is required for the efficient recycling of CO_2_ in our bodies. The Bio‐Field Array (BFA) shows potential to both drive/fuel the zeta potential and restore the Golden Ratio in human erythrocytes thereby leading to more efficient recycling of CO_2_. Live Blood Analyses and serum CO_2_ levels from twenty human subjects that participated in immersion therapy sessions with the BFA for 2 weeks (six sessions) were analyzed. Live Blood Analyses (LBA) and serum blood analyses performed before and after the BFA immersion therapy sessions in the BFA pilot study participants showed reversal of erythrocyte rheological alterations (per RBC metric; *P* = 0.00000075), a morphological return to the Golden Ratio and a significant decrease in serum CO_2_ (*P* = 0.017) in these participants. Immersion therapy sessions with the BFA show potential to modulate zeta potential, restore this newly defined Golden Ratio and reduce rheological alterations in human erythrocytes.

## Introduction

**Figure 1:**
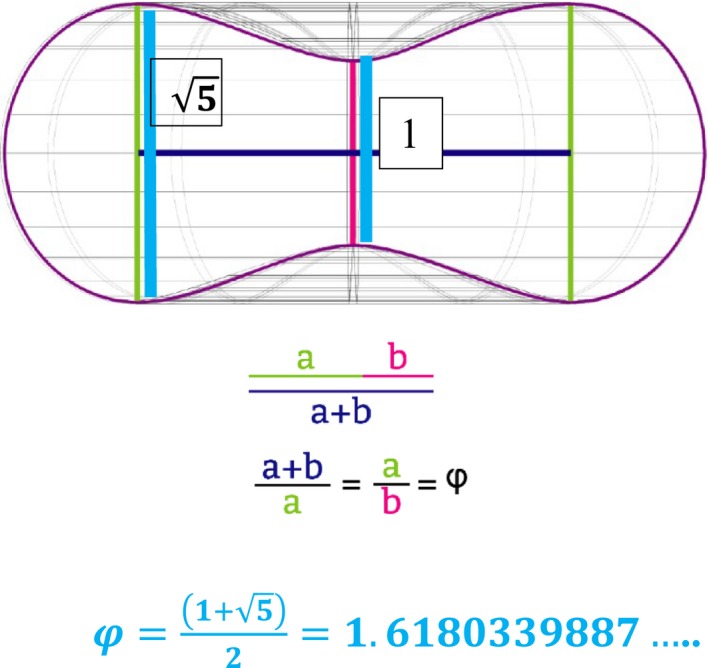
The Golden Ratio Geometrical Measure of the Erythrocyte that represents the DEP EMFFF: Upon cross‐sectional analyses – the sum of the quantities to the larger quantity (**a  +  b**) is equal to the ratio of the larger quantity (**a**) to the smaller one (**b**) represent the entire Golden Ratio area of the Erythrocyte; And a measure of one representative radius is represented in this equation:‐‐> $$\varphi =\frac{\left(1+\sqrt{5}\right)}{2}=1.6180339887\ldots$$Lensless microchip sensor capabilities can be developed to measure this Golden Ratio proportion for a potential new biomarker in medicine. The average diameter of a human erythrocyte is 6.2–8.2 *μ*m with the thickest point being 2–2.5 *μ*m ($\sqrt{5}$) and the minimum thickness at the center of 0.8–1 *μ*m (1) divided by 2 (for the two equal and opposing sides of the proportion) to achieve the 1.6803339887 or the golden ratio geometric proportion of the erythrocyte. Measurement of the center depth of the torus and at the thickest point of the torus divided by 2 represents *a measurable relative radial proportion* of the Golden Ratio in the RBC. There is a division of 2 in the equation to account for an equal and opposite geometric measure of the Golden Ratio across the toroid (as seen in a/b proportions). This measurable relative radial proportion would then be set into normal range values for the RBC Golden Ratio (i.e., 1.40–1.75).

**Figure 2:**
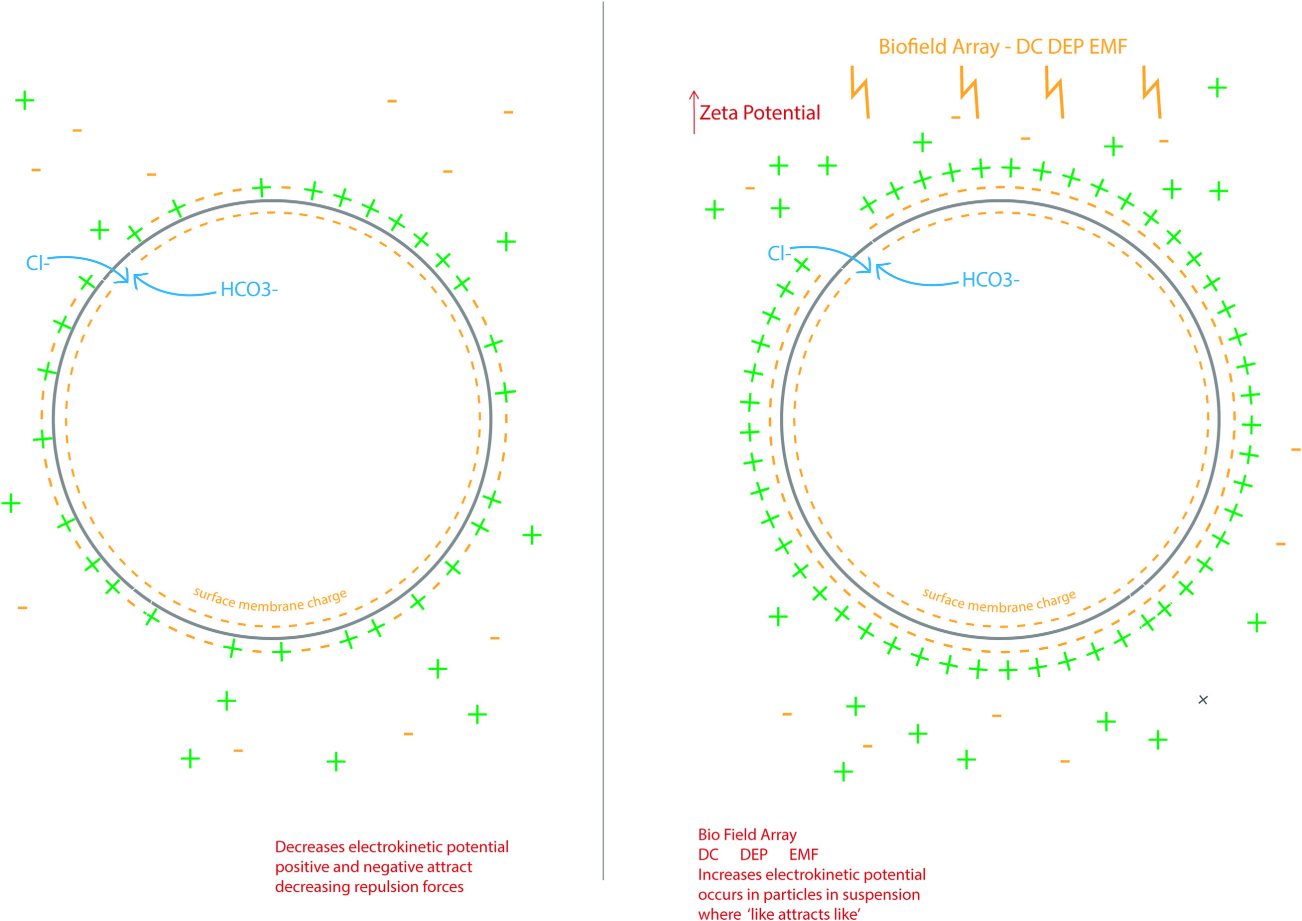
Zeta Potential/Electrostatic Field of the Erythrocyte Torus. While membrane potential can be measured *across* most other plasma cell membranes, the external field of a torus is actually measured as zero *on* the surface of the plasma membrane (Papasimakis et al. 2016). This current has been demonstrated in the widely known zeta potential phenomena where the dielectric constant (chloride) separates the negative surface membrane charge on the erythrocyte from the positively charged Stern layer of the serum to create a static flow (separation of the negative surface membrane charge from the Stern layer on the right) on the torus surface. The BFA dc DEP‐EMF has been shown to modulate ferroelectric changes in the chloride ion and chloride ion channel expression (Purnell and Skrinjar 2016b). This Zeta Potential/Electrostatic Field may drive the center DEP EMFFF in the erythrocyte (Figure 3). The center DEP EMFFF may be essential in the recycling of CO_2_ (when combined with H_2_O) into H^+^ and HCO3^−^. In the peripheral circulation, a bicarbonate (HCO_3_‐) leaves through Band 3/AE1 and a Cl‐ spins into the cell to retain cell neutrality.

**Figure 3:**
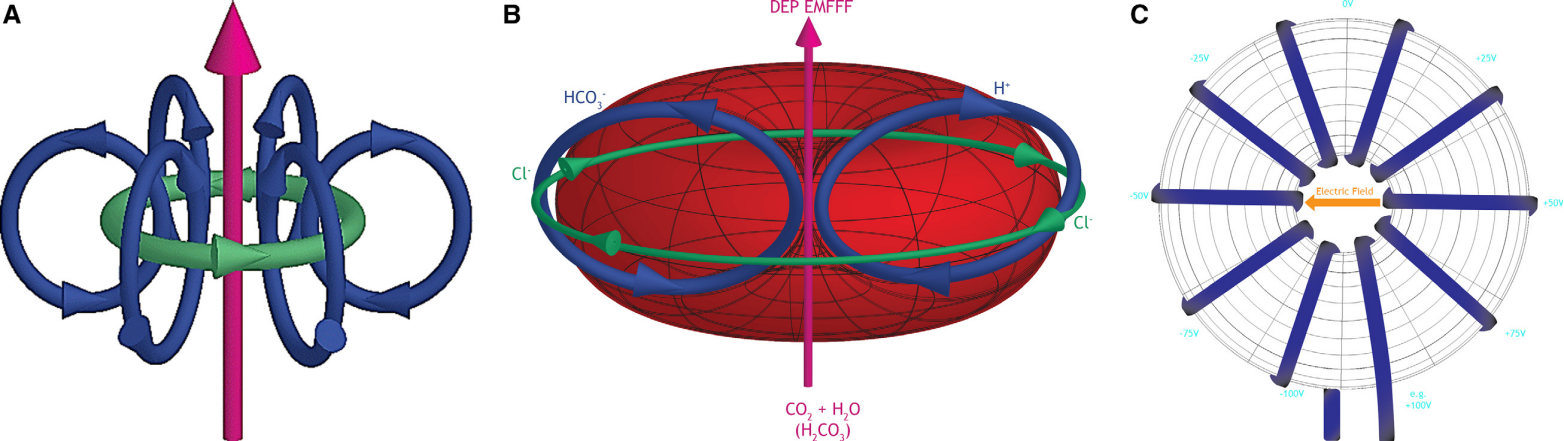
Toroidal Flow of the Erythrocyte with Dielectrophoretic Electromagnetic Field Flow Fractionation (DEP EMFFF) (A). Magnetic toroidal flow of the erythrocyte that is fueled by the electrostatic field/zeta potential on the membrane surface. The DEP EMFFF particles (H_2_O + CO_2_) enter into the flow that passes through the separation chamber (positive and negative flow around the toroid‐ **green arrows**) with external separating forces being applied perpendicular to the flow (multipole spin‐ **blue arrows**) (B). Erythrocyte toroidal flow with red arrow pointing to DEP EMFFF in the area of the Golden Ratio seen in Figure 1. The static toroidal dipoles (driven by the external static current/zeta potential‐ Figure 2) create the internal magnetic DEP EMFFF that is confined within the Golden Ratio proportion of the red blood cell torus and does not interact directly with this external (zeta potential) electrostatic field. This DEP EMFFF, through hydrodynamic (water) and dielectric (Cl‐) influences, separates H_2_ CO_3_ into the positively charged H^+^ which flows to the membrane surface to be used to make (hemoglobin) Hg to carry O_2_ on the toroid surface and the negatively charged (bicarbonate) HCO_3_^−^ which exits into the plasma (through anion channel Band 3/AE1) for use acid–base control in the body (while a chloride ion (Cl‐) enters the cell. Parabolic velocity of the flow causes the HCO_3_^−^ to move further away from the cell membrane to be eluted from the cell at a faster rate as opposed to H^+^ that remains in the membrane. (C). E‐field of a simple toroid (+/‐ volt excitation).

**Figure 4:**
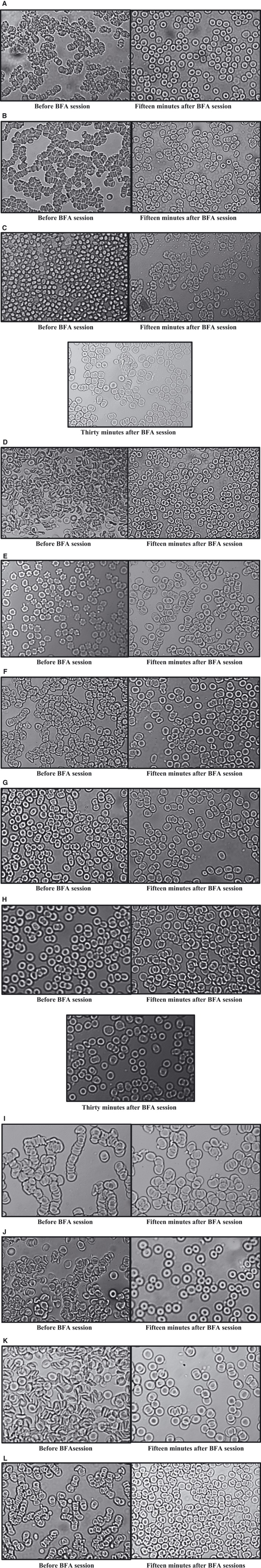
Demonstration of the Rheological Alterations via Live Blood Analysis before and after BFA Immersion Therapy Sessions in study participants. Note the change to more single biconcave discs in the after pictures. All pictures were taken immediately before the BFA sessions and 15–30 min after the BFA sessions. (A) Before BFA Immersion Therapy Session, the LBA shows the rheological changes (rouleaux, clumping) and oxidative stress on the cell membranes. The after BFA Immersion Therapy Session LBA shows progression to biconcave discs with a defined center neutral (Golden Ratio region shown in Fig. 1). (B) A before BFA Immersion Therapy Session LBA shows rheological changes of rouleaux and clumping. The after BFA session LBA shows a decreased in the rouleaux, a defined center neutral center (Golden Ratio region shown in Fig. 1) and a progression to individual biconcave discs. (C) A before BFA Immersion Therapy Session LBA in this participant shows rheological alterations in size, shape and geometric proportion of the erythrocytes in this participant. Fifteen minutes after the BFA session, the LBA shows return to appropriate size, shape proportion of the erythryocytes with some loose rouleaux chaining occurring. Thirty minutes after the BFA session the LBA shows progression to individual biconcave disc formations. (D) A before BFA Immersion Therapy Session LBA shows rheological alterations of clumping, rouleaux, along with shape and proportion changes. The after BFA session LBA shows a progression to the biconcave disc shape. (E) A before BFA Immersion Therapy Session LBA shows size and shape changes in the erythrocytes. The after BFA session LBA shows a return to the appropriate biconcave disc shape with some loose rouleaux chaining. (F) A before BFA Immersion Therapy Session LBA shows rheological alterations of rouleaux, size, shape and proportion. The after BFA session LBA shows a reduction in rouleaux, increased size and progressing to the appropriate biconcave disc shape. (G) A before BFA Immersion Therapy Session LBA shows loose rouleaux and some mild clumping. The 15 min after BFA Session LBA shows some mild reduction in rouleaux and clumping. (H) A before BFA Immersion Therapy Session LBA shows no apparent rheological alterations. The fifteen minute after BFA session LBA shows some mild chaining. The thirty minute after BFA session LBA (after participant was encouraged to drink some water) showed an increased erythrocyte ‘current’ movement on the slide and a return to the biconcave disc shape. (I). A before BFA Immersion Therapy Session LBA shows rouleaux and the 15 min after BFA session LBA shows a reduction in this rheological alteration and progression to biconcave discs. (J) A before BFA Immersion Therapy Session LBA shows rheological alterations of rouleuax and clumping along with size, shape, and proportion changes. The after BFA session LBA shows a return to biconcave disc shapes with a well‐defined center/Golden Ratio area. (K) A before BFA Immersion Therapy Session LBA shows rheological alterations of rouleaux, chaining and clumping. The after BFA Session LBA shows progression to the biconcave disc shape with some very mild loose chaining. (L) A before BFA Immersion Therapy Session LBA shows rheological alterations of rouleaux and chaining. The after BFA Session LBA show a reduction in the rouleaux and chaining with an apparent decreased serum noted around the blood cells.

**Figure 5:**
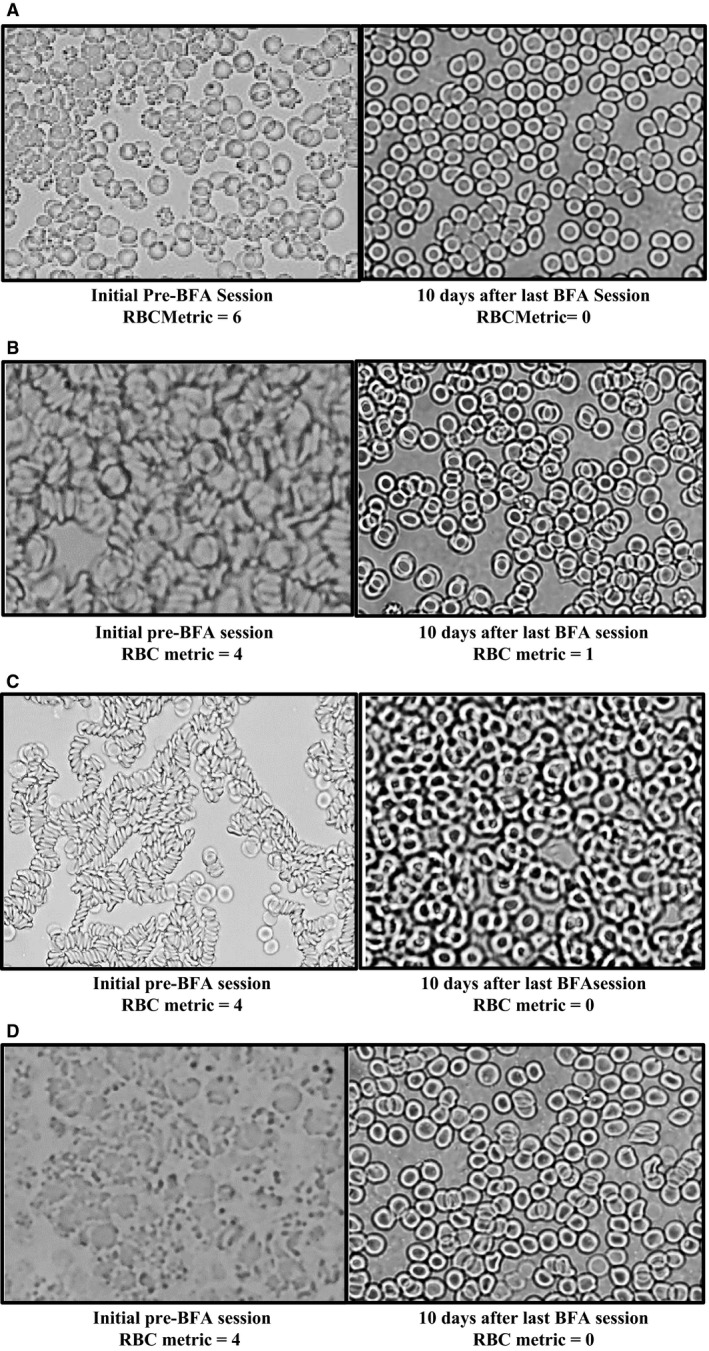
LBA changes (RBC Metric scores) noted from the Initial LBA taken prior to the first BFA Session in the study participants and the Final visit LBA which was taken 7–10 days after the end of the 2 week study (After 6 BFA Immersion Therapy Session visits). The participants did not have a BFA session prior to the Final visit LBA.

The Golden Ratio is an irrational number and a function of Phi *φ*, and has been studied by mathematicians, biologists, artists, musicians, historians, architects, etc. for centuries (Livio 2008). Marcus Vitruvius Pollio, a Roman architect and author of the multi‐volume work *Be Avehiteetuve* (c. 25 B.C.), commented on the similarity between the human body and a perfect building: “Nature has designed the human body so that its members are duly proportioned to the frame as a whole” (Abu‐Taieh 2015). This irrational number is represented in the formation of all living things including the size, shape, proportions and curvature of the erythrocyte (Zhang & Ou‐Yang, 2016). To date, the importance of the size, shape, proportions and curvature of the red blood cell that constitute the Golden ratio has often been underestimated. It appears that this living geometry of the red blood cell may be critical for efficient oxygen/carbon dioxide exchange and balance in the body (Fig. 1). Scientists are now beginning to understand that many problems in biomedicine can be linked to the control of complex biological shape with regards to geometric changes that can be seen in embryogenesis, traumatic injury, degenerative changes and cancer (Levin 2013). Also, since the red blood cell is sensitive to oxidative alterations, the erythrocyte morphology is often the first to be affected by these alterations and is often used as a first‐step diagnosis in a number of pathologies (Pandy and Rizvi 2011). The erythrocyte is highly specialized and the most abundant cell in the body which makes it an interesting cell to study since it differs in many ways from other eukaryotic cells in that it has no internal membranes and the surface membrane appears to operate very differently from other cell types (Pandy and Rizvi 2011). The plasma and internal membranes of other eukaryotic cells can be considered to function like a dielectrophoretic electromagnetic dipole where anions and cations act as a current loop to drive membrane potential on either side of the cell membrane (Purnell and Skrinjar 2016a,b). The red blood cell unique design is a toroid where currents also flow on the surface of the torus (Fig. 2) (Papasimakis et al. 2016). When this surface current flow is static (due to efficient separation of the positively charged Stern layer and negative surface membrane charges; Fig. 2), the zeta potential is enhanced and the size, shape, proportion, curvature of the erythrocyte transforms to a Golden Ratio proportion (Figs. 1, 2, 3).This Golden Ratio proportion of the red blood cell appears to house a dielectrophoretic electromagnetic field flow fractionation (DEP EMFFF) that may participate in the efficient delivery of O_2_ (through hemoglobin production and function) to the tissues and just as importantly to recycle the cellular respiration waste product of CO_2_ in our bodies. The static current (separation of charges with a dielectric constant) on the surface of the toroidal dipole (Fig. 2A and B) are essential to create the DEP EMFFF that is confined within the center neutral zone of the torus, forms the Golden ratio and does not interact directly with this external field (Figs 1, 2). (Papasimakis et al. 2016). The interplay of both electric and magnetic (multiferroic) orders in metal‐organic frameworks has intrigued biophysicists and has led to a pursuit of a new class of multiferroics beyond the current solid‐state or electronic applications (Fiebig et al. 2002; Kimura et al. 2003; Cheong and Mostovoy 2007; Tian et al. 2014; Qi et al. 2015). Multiferroic materials are known to modulate both magnetic and electric orders such as: ferromagnetism (a spontaneous magnetism that is switchable by an applied magnetic field), ferroelectricity (a spontaneous electric polarization that is switchable by an applied electromagnetic field) occurring in the same phase (Hur et al. 2004; Spaldin and Fiebig 2005). The BFA applies both electric and magnetic signals and appears to be a novel multiferroic application that exhibits both ferromagnetic and ferroelectric changes in and around cellular membranes of living organisms (Hur et al. 2004; Purnell and Skrinjar 2016b). When this static flow on the erythrocyte membrane surface is interrupted by a lack of separation between the negative surface membrane charge and the Stern layer, the zeta potential weakening leads to geometric proportion distortion, decreased electric permittivity, increased viscosity, flocculation and rheological alterations (Figs. 2A, 4, 5). When these known rheological alterations and distortion of the Golden Ratio proportion occur, there is a decreased efficiency of O_2_/CO_2_ exchange possibly due to a disruption in the DEP EMFFF that resides in the center of the torus (Figs. 1, 2, 3, 4, 5).

A critical component that is critical to transfer a solid‐state science to organics is to incorporate and use water. Water has an extraordinary ability to both shield and enhance/amplify different charged species, has a high dielectric constant (ԑ) and is the matrix of all known living organisms (Kurian et al. 2017). Nobel Prize winner Montaignier has shown that water clusters (nanoparticles) are able to transmit electromagnetic signals between each other and the outside world (Montagnier et al. 2009). The Bio‐Field Array is a device that delivers ~2.5 to 3 amperes of direct current (dc) to an array that consists of a series of conductive rings that are designed to create a dielectrophoretic (DEP) electromagnetic (EMF) field in the water or a hypotonic ionic solution (Purnell and Skrinjar 2016a,b; Purnell 2017).

In our recent BFA pilot and feasibility study, we conducted Live Blood Analyses (LBA) to observe rheological alterations of the human erythrocyte in study participants (Figs. 4, 5). Rheological alterations are currently linked to morbidities such as: diabetes, hereditary disorders, sickle cell, myocardial infarction, paroxysmal nocturnal hemoglobinuria, sepsis, end stage renal disease, hypertension, strokes, endothelial function, vascular health, hemostasis in athletes and blood transfusions and cancer (Hung et al. 1991; Pechan et al. 1991; McHedlishvili and Maeda 2001; Ahmad and El‐Sayed 2003; Baskurt and Meiselman 2003, 2010; Piagnerelli et al. 2003; Babu and Singh 2004; Meiselman et al. 2007; Forsyth et al. 2012; Serroukh et al. 2012; Beck et al. 2014; Giovanna 2014; Hierso et al. 2014; Buttari et al. 2015; Heber and Volf 2015; Ertan et al. 2017). Interestingly, we also observed many rheological alterations in our “healthy” pilot study participants (Figs. 4, 5). The Bio‐Field Array (BFA) appears to reverse rheological changes and possible restore and maintain the Golden Ratio in human erythrocytes. (Papasimakis et al. 2016; Zhang & Ou‐Yang, 2016). The focus of this paper is to further examine methods and concepts to help define and explain these red blood cell changes noted in the original BFA pilot and feasibility study we conducted. Since our cell membranes have been described as liquid crystal semiconductors, the ability to utilize multiferroic applications through the coupling of magnetic and electric influences to promote cell membrane modulation may lead to further understanding of ways to affect cell physiology and ultimately address both chronic health issues as well as prevention and wellness in medicine (Lipton 2008).

## Methods

Twenty healthy study participants (10 male/10 female ages 18–34) were enrolled in an Institutional Review Board (IRB) approved pilot and feasibility study (protocol number: PRO‐FY2017‐9) performed at the University of Memphis, Memphis Tennessee. All study participants were recruited from the University of Memphis student population, screened for eligibility using defined inclusion and exclusion criteria and signed an IRB approved informed consent providing their consent to participate in the study. Each study participant had a physical examination, 12‐lead electrocardiogram [ECG], urinalysis, pregnancy tests for females, Complete Metabolic Panel [CMP], Complete Blood Count [CBC], Live Blood analysis [LBA], and Sedimentary Rate [Sed Rate] performed before the first BFA treatment and the 1 to 2 weeks after the last BFA treatment. Protocol and data collection forms were used to acquire demographic and medical information. A board certified physician performed the physical examinations and reviewed the EKG and blood work results. Additional quantitative and qualitative data were collected by requiring study participants to complete the PROMIS v1.1 Global Health questionnaire and a life diary during the study (Baumhauer 2017).

### Bio‐field array treatment

The Bio‐Field Array device (BFA) that consists of a power supply that converts alternating current (AC) to DC was used in this pilot and feasibility study. The BFA device uses 2.5 to 3 amperes of direct current applied to an array that consists of seven specifically designed and spaced conductive and nonconductive metal rings which is placed in a hypotonic saline solution and delivers a DEP EMF to the saline solution. Eight liters of warm tap water were placed in a 12‐liter plastic washbasin and one to two teaspoons of Himalayan salt were added to achieve 2.0–2.5 amperes of conductivity in the water (~3 mmol/L hypotonic ionic solution) as measured by the ampere meter on the power supply. The tap water used in the trial originated in the city of Memphis, Tennessee. Water qualities across the globe have historically required different amounts of salt to achieve this recommended ampere setting with this BFA device. The participants then sat in a chair and placed their feet in the washbasin with the warm hypotonic saline solution and the BFA for a 35‐min treatment with active DC running to the BFA. The participants came in 3 days a week (every other day) for 2 weeks for a total of six 35‐min BFA immersion sessions.

### Live blood analyses

Live Blood Analyses were conducted as a substudy in the BFA pilot and feasibility study and were performed on 18 of the 20 participants immediately prior to and after their initial Bio‐Field Array immersion therapy sessions and 7 to 10 days after their completion of the 35‐min Bio‐Field Array sessions. To collect the blood sample, we first cleaned the participant's fingertip with alcohol and then used HTL‐Strefa Acti‐Lance Safety lancets and conducted their finger sticks. The first drop of blood was wiped away to ensure all the alcohol was removed from the sample and the second drop of blood was placed onto a slide and immediately covered with a coverslip. The blood sample was immediately analyzed for erythrocyte morphology, oxidative stress, platelet aggregation and rouleaux or chaining under 100X microscopy. Pictures of each sample were taken for comparison. LBA can be analyzed using dark‐field, phase contrast and bright‐field microscopy. Bright‐field microscopy was used in the study because it is the simplest of all microscopy illumination techniques and could be easily applied in clinical practice (Kirby and Hasselbrink 2004). Live blood analyses allows for observation, in real time, of the size, shape and curvature of the red blood cells, presence of other rheological alterations and aggregation of thrombocytes or platelets and could be a potential indicator of potential thrombosis and possible impending stroke or heart attack and these phenomena would be disrupted if the cells were processed prior to observation (most anti‐clotting agents in processed blood today greatly affect these observations) (Rauf 2013). Since hydration plays a role in the ionic strength of the plasma (zeta potential) the study participants were also asked to drink a 12 oz. bottle of Ozarka Spring water.

### Red blood cell (RBC) metric analyses

In order to document and compare changes in the live blood samples in the study participants in the trial a scoring system was developed (Table 1). The parameters of interest were: (1) erythrocyte morphology; (2) oxidative stress (presence of Heinz bodies); (3) number of erythrocytes in rouleaux chaining. Erythrocyte morphology was graded by assigning 0–2 points for: (1) round/oval symmetrical biconcave discs = 0 points; (2) irregularly shaped biconcave discs = 1 point; (3) irregularly shaped cells lacking biconcave morphology = 2 points. Oxidative stress was graded by assigning 0–2 points for: (1) No Heinz bodies or other plasma components adhered to or visualized sticking to the red blood cell membrane = 0 points; (2) serum components visualized sticking to the red blood cell membranes = 1 point; (3) serum components visualized sticking to the red blood cell membranes and red blood cells also beginning to aggregate/clump = 2 points. Rouleaux or chaining was graded by calculating the number of RBCs in chains: (1) no chaining of red blood cells noted = 0 points; (2) less than 4 red blood cells found in rouleaux/chaining = 1 point; (3) greater than 4 red blood cells found in rouleaux/chaining = 2 points. A score of 0 equals individual red blood cells with the round biconcave disc shape are noted, while a score of 1–6 denotes rheological alterations and a dysfunction in the Golden Ratio are present in the erythrocytes (Table**** 1). Paired t tests of the RBC Metric Analyses were conducted on the participants' initial live blood analysis (before their first BFA session) and at their final clinic visit live blood analysis (Table 2). The final participant study visit was conducted ~7–14 days after their last BFA treatment that was completed at the research subject's sixth visit.

**Table 1:** Red blood cell (RBC) metric

Parameter	0 points	1 point	2 points
Erythrocyte Morphology	RBCs are biconcave discoids	RBCs are biconcave but not discoid‐shaped	RBCs are not biconcave or discoid‐shaped
Oxidative Stress	No Heinz bodies adhered to the RBCs	Heinz bodies are Adhered to the RBCs with no clumping	Heinz bodies are adhered To the RBCs with clumping
Rouleaux	No chaining of RBCs	Chains with less than 4 RBCs	Chains with more than 4 RBCs

**Table 2:** Paired t tests with RBC Metric from Initial Live Blood Analysis and Final Visit Live Blood Analysis in 18 Feasibility Study Participants with a score 0 representing lack of rheological alterations and scores of 1–6 representing rheological alterations and most likely Golden Ratio distortion

E/P Metric	Paired *t*‐Tests			Mean/Initial	Mean/Final
	df	ts	*P*		
Initial/Final	17	7.583431	0.00000075	3	0.3889

### Statistical analyses

Paired t tests were conducted on the developed RBC Metric analysis of each participant for comparison between the means of the first LBA prior to the participant's first BFA treatment session and the final LBA, which was conducted 1–2 weeks after with final BFA treatment. Serum CO_2_ values were analyzed with B = 200 bootstrap replications to detect a difference between two mean functions in the setting of nonparametric regression.

## Results

Rheological alterations in red blood cells such as rouleaux, oxidative stress, and other morphological changes were noted in the LBA conducted in the participants prior to their initial BFA immersion therapy sessions with progression to the desired biconcave disc shape in their LBA after these sessions (Fig. 4). Rouleaux/chaining and oxidative stress was observed on the plasma membranes of the erythrocyte in a study participant (Fig. 4A). After a 35‐min BFA immersion therapy session, the rouleaux/chaining and membrane stress appear to progress to biconcave discs. Rouleaux/chaining that progressed to biconcave disc shaped cells is noted after the BFA session (Fig. 4B). An interesting phenomena of smaller than expected red blood cells with unique morphology changes was noted on the erythrocyte membranes before the BFA immersion therapy session and then showed return to desired cell size, shape and proportion (with loose chaining) after the 35 min BFA session (at the 15 min postsession time point) (Fig. 4c). This participant was then encouraged to complete the drinking of a bottle of Ozarka Spring water (12 oz.) and the LBA was then conducted at the 30 min time point post‐BFA session with a further progression to the biconcave disc shape (Fig. 4C). Red blood cells with rouleaux, chaining, shape and curvature changes that progressed to the biconcave disc morphology also occurred after the BFA 35 min session (Fig. 4D). Size and shape changes were noted and after the BFA immersion therapy session, there is a return to the desired size and shape with some loose chaining (Fig. 4E) while Figures 4F, show progression to biconcave disc shape. Figure 4H shows some initial loose chaining 15 min after the BFA treatment and a return to the biconcave disc shape at the 30 min time point after treatment and after drinking the spring water as well. Figure 4i shows an interesting progression to a more defined center in the Golden Ratio proportion area noted in Figure 1. There were significant microscopic differences noted in a few of the study participants upon their initial visit LBA when compared to their final visit LBA (Figs. 5a–d).

Due to the unexpected LBA results, we developed a Red Blood Cell (RBC) Metric (Table 1) that can be quickly used (via light microscopy) to screen for rheological alterations and Golden Ratio proportion dysfunction using LBA. We analyzed the red blood cell morphology, oxidative stress (presence of Heinz bodies on the RBC surface) and rouleaux using our developed RBC Metric (Table 1). There was a significant difference in the initial LBA RBC metric mean (M = 3) and final visit LBA RBC metric (M = 0.3889) in the BFA study participants when analyzed with paired *t*‐tests (Table 2).

Lastly, the CMP laboratory values that were measured in the study participants prior to their first BFA treatment in the screening visit and at the Final visit after their last BFA study visit number 6 (where their last treatment was conducted) showed a significant drop in the mean serum CO_2_ (*P* = 0.017; Table 3). It is interesting to note that the significant drop approached the median of the normal range with the mean CO_2_ of the participants residing closer to the upper normal limits of the CO_2_ range at the initiation of the study. There may be a correlation of a significant decrease in the rheological alterations via the developed RBC Metric (Table 1, 2, 3) and the significant decrease in mean serum CO_2_ in the study participants with both measured at screening and final visits.

**Table 3:** Mean serum CO_2_ levels at screening visit and final visit

Serum CO_2_	B = 200 Bootstrap Replications			Mean/Initial	Mean/Final
	*n*	SE	*P*		
Initial/Final	20	1.132	0.017	26.50	25.30

## Discussion

### Ferromagnetic and ferroelectric (Multiferroic) influence on zeta potential

The erythrocyte can be considered a spherical *capacitor* of the body and surface area on a capacitor is very important with regard to its efficiency (Ho et al. 2010). This cell's membrane surface must maintain a static current flow to remain free of other erythrocytes, platelets, oxidative proteins, etc., in order for the optimal surface membrane exchange of oxygen (O_2_) for carbon dioxide (CO_2_) to occur in the body (Fig. 2, 3, 4, 5). Band 3/AE1 is an anion channel that appears to be gated by the chloride ion. The BFA dc DEP‐EMF may induce a ferroelectric change (polarity) in the chloride ion that not only acts as a dielectric constant to separate the charges on the membrane surface to create the static current flow, but also gates Band 3/AE1 (Fig. 2). If the cations in the plasma are interfering with or occupying the space at the negative membrane surface area, the Cl‐ is not able to adequately surround the membrane in order to be readily available to conduct an exchange for HCO3 – in order to maintain cell neutrality as it exits the cell (Fig. 2). An interruption in the field driven by chloride polarity, ionic strength of the serum, and serum iron (anemia) may then decrease/weaken the zeta potential thereby leading to undesired rheological alterations and a deformation of the Golden ratio proportions (Figs. 2, 3). The zeta potential is a good measurement of the electrical repulsive forces between particles as a function of distance. The zeta potential is also the critical control mechanism that offers stability of colloidal dispersions such as blood plasma and prevents flocculation and ultimately these observed rheological alterations (Kirby and Hasselbrink 2004). The Zeta potential (*ζ*) (Equation (1)) equals the ionic strength (viscosity) of the medium (*ŋ*), electronegative charge of the RBC membrane (electrophoretic mobility) (*μ*), divided by the dielectric constant (*ԑ*).

(1) ζ=4πη(μ)/(ε)

The calculation of the zeta potential, using the Smoluchowski Equation depicts electrophoretic mobility (*μ*) as equal to electric permittivity of the liquid (*ԑ*) divided by the viscosity (*ŋ*) of the plasma (Equation (2)). This is applicable when there is a thin double layer, stable zeta potential and with large colloidal particles (RBCs) and high ionic strength. Video S1 displays an increased electrophoretic mobility of the red blood cells after a BFA immersion therapy session in a study participant when compared to the red blood cells in Video S2 that was taken prior to the BFA immersion therapy session in a study participant. We propose that this increased electrophoretic mobility noted in Video S1 may occur due to the enhanced dielectric constant (ferroelectric change in chloride on the RBC membrane surface), increased electric permittivity (causing a vacuum to surround and shape the erythrocyte) and decreased viscosity of the RBC microenvironment.

(2) μ=ϵξ/η

Upon analysis of the LBA in the study participants, we observed some rheological alterations after the BFA immersion sessions on occasion that were reversed with the addition of water intake. This highlights the importance of the previously known ionic (dilutions) strength (*ŋ*) portion of the zeta potential equation. Once there is a ferroelectric and ferromagnetic influence (from the BFA‐dc‐DEP‐EMF) on and around the surface membrane charge, dielectric constant, and the ionic strength of the plasma, the erythrocyte will repel other erythrocytes (and other plasma components). Essentially, the negatively charged chloride anions are diamagnetic and may now attract the diamagnetic hydrophilic region in the erythrocyte membrane (due to glycophorins) and are separated/repelled by the cations in the positively charged Stern layer (Fig. 2). If a red blood cell loses this essential separation of negative surface membrane charge and positive Stern layer charges by a drop in zeta potential (Fig. 2) due to factors affecting the ferromagnetic serum iron and the ferroelectric chloride ion influence, and/or a change in the ionic strength of the serum (i.e., electrolyte imbalances), the erythrocytes will not only begin to attract each other and other plasma components (rheological alterations occur), but will ultimately display distortions of their Golden ratio proportions (due to loss of the electrical permittivity vacuum) as seen in the before BFA immersion therapy session figures (Figs. 4, 5). These rheological alterations that are linked to multiple chronic comorbidities may be due to the distortion of the Golden Ratio proportions and weakening of the center dielectrophoretic electromagnetic field flow fractionation (DEP EMFFF) that impairs and reduces the recycling of CO_2_ (Figs. 1, 2, 3, 4, 5). It is important to note that many will say that iron only exists in the body as a weak paramagnetic ion. We propose that this must be rethought in the terms that ferromagnetism is a magnetization that is switchable by an applied field. The magnetization appears to switch in the study participants that exhibit changes in their erythrocyte morphology after the removal of the BFA's active field for a currently unknown period of time (we measured 7–10 days after their last BFA treatment) (Fig. 5). This lasting effect correlates with a possible ferromagnetic influence (serum iron), which is a magnetism that exists after the removal of an applied field. The BFA dc DEP‐EMF appears to influence changes in size, shape, proportion, and contour of the erythrocyte (Figs. 4, 5) that display the Golden Ratio, which may represent zeta potential changes in the external field of this toroidal cell.

### Dielectrophoretic electromagnetic field flow fractionation

A DEP EMFFF in the center of the erythrocyte is contained within the Golden ratio geometric proportions of this cell and may be essential for the recycling of the by‐product of cellular respiration, CO_2_, in our bodies (Figs. 1, 3). Erythrocytes have a diameter of 6.2–8.2 *μ*m, are 2–2.5 *μ*m at the thickest point, and are at least 0.8–1 *μ*m thick in the center (Fig. 1). These measures and the Golden Ratio proportions we developed can be seen in Figure 1. This DEP EMFFF, through hydrodynamic (water) and dielectric (Cl‐) influences, separates H_2_CO_3_ into the positively charged H^+^ which flows to the membrane surface to be used to make (hemoglobin) Hg to carry O_2_ on the toroid surface and the negatively charged (bicarbonate) HCO_3_^−^ which exits into the plasma (through anion channel Band3/AE1) for use in the acid‐base balancing of the body (while a Cl‐ enters the cell) (Fig. 3) (Davis and Giddings 2006). Parabolic velocity of the flow causes the HCO_3_^−^ to move further away from the cell membrane to be eluted from the cell at a faster rate as opposed to H^+^ that remains in the membrane (Fig. 3). Approximately, 70–75% of the CO_2_ in the plasma diffuses across the erythrocyte lipid bilayer membrane (through aquaporin channels) for recycling and this diffusion is facilitated by the nonpolar nature of the CO_2_ molecule (Fig. 3) (Rice et al. 2014). The CO_2_ then combines with a H_2_O molecule to become H_2_CO_3._ When this H_2_CO_3_ molecule enters the center DEP EMFFF of the erythrocyte, it disassociates into a proton H^+^ (makes hemoglobin and resides on the surface of the red blood cell to carry O_2_) and HCO_3_^−^ (bicarbonate) to help with acid–base homeostasis (Fig. 3). The other 25–30% of the CO_2_ remains available in the plasma to be used by the lungs for regulation of the acid–base homeostasis (Rice et al. 2014). This internal DEP EMFFF is fueled/driven by the zeta potential's influence on the toroidal flow on the membrane surface but does not interact with the external field (Fig. 3). This DEP EMFFF disassociates positive and negative charged ions around the E‐field of the toroid (Fig. 3) (Purnell and Skrinjar 2016b). The neutral carbonic acid, H_2_CO_3,_ is separated/recycled once it enters the DEP EMFFF into cations (H^+^) and anions (HCO3^−^) (Fig. 3). While chloride performs the function of the dielectric constant in the zeta potential, it also functions as the gate keeper to the Band 3/AE1 anion channel. Chloride spins at the membrane surface and as HCO_3_‐ exits the toroidal DEP EMFFF, a chloride ion spins into the torus red blood cell (as bicarbonate leaves), thereby maintaining intracellular neutrality (Figs. 2, 3) (Purnell and Skrinjar 2016b).

We have identified the geometric proportion of the Golden Ratio of the erythrocyte (Equation 3) that is based in the irrational number Phi (*φ*) (Fig. 1). These identified proportions can be used by clinicians as a new biomarker (with imaging a droplet of blood) to determine the efficiency of the erythrocyte in both the recycling of CO_2_ and the O_2_ carrying ability (Fig. 1).

(3) $$\varphi =\frac{\left(1+\sqrt{5}\right)}{2}=1.6180339887\ldots$$

To date, the altered/impaired magnetic attraction of the ions on the erythrocyte surface and how to restore the Golden Ratio that appears in the red blood cell shape has remained unclear (Kim et al. 2015). Carbonic Anhydrase has been thought to be the enzymatic catalyst that separates H_2_CO_3_ into H^+^ and HCO_3_^−^ and has been a topic of research across the globe for use as biomarkers in hypoxia, for prognostic purposes and for predictive use in therapeutic trials in various cancers (Wind et al. 2011). We propose that the negatively charged diamagnetic chloride anions and diamagnetic hydrophilic region of the plasma membrane interact to form an interfacial exclusion zone between the negative surface membrane and the adjacent positively charged Stern layer of the erythrocyte that allows for a separation and exchange of charged ions (static current that drives zeta potential) that is fueled by the BFA dc‐DEP‐EMF and drives the center DEP EMFFF for the efficient recycling of CO_2_ (Pollack et al. 2009; Purnell and Skrinjar 2016b). The Golden Ratio proportion that may be essential for an effective DEP EMFFF that is required to recycle the waste product of our cellular respiration can now be measured due to our identification of the Golden Ratio of the red blood cell in Figure 1. This can be calculated and measured with range values as a laboratory value much like other serum laboratory values are measured today. We currently fully understand the acute elevations of CO_2_ in the body and the health crisis that it can create. From observation of the LBA in these young healthy study participants, rheological alterations occur frequently in this seemingly healthy population. The body is a master compensator and these unnoticed rheological alterations in the erythrocytes and mildly increased CO_2_ will most likely predispose these individuals to chronic disease such as hypertension, arrhythmias, etc. in the future (Shoemaker et al. 2002). We may have neglected the importance of chronically and mildly elevated CO_2_ levels in the young (and others) along with the repercussions they may have on their futures states of health. Since these rheological alterations are strongly linked in the literature to multiple health pathologies, understanding, defining, measuring, and actually treating these alterations and restoring the Golden Ratio may indeed have significant impacts on health and wellness.

## Conclusion

The anion (Cl^−^ and HCO3^−^) transport capacity of the erythrocyte membrane is one of the largest of any cell membrane (Wieth et al. 1982). This BFA feasibility trial suggests that the ability to modulate the dielectric constant (chloride ion) of the medium/plasma with ferroelectric changes on the erythrocyte membrane surface (essentially a zero charge on the membrane surface) may be occurring and may also be a novel intervention that can reverse or reduce common rheological alterations, restore the Golden Ratio driven DEP EMFFF and significantly lower serum CO2 levels (*P* = 0.017) in these healthy human subjects. The ability to modulate chloride ions and chloride ion channels has remained elusive to date and unfortunately, tools to measure a ferroelectric change in a molecule such as chloride have yet to be developed. It appears that the BFA dc‐DEP‐EMF's electric and magnetic influences could be a novel multiferroic application in medicine. The erythrocyte displays a very unique dielectrophoretic toroidal dipole field that appears to be maintained by the BFA dc‐DEP‐EMF multiferroic influence. When this field is weakened, there may be a decreased toroidal excitation (and zeta potential) that leads to the loss of the Golden Ratio proportion with an increased occurrence of rheological alterations. It is time we begin to both give credence to and understand how this Golden Ratio could offer a mechanistic explanation for not only the beautiful design of this unique cell but also the potential for a notable biomarker that could predict the development of chronic disease. Miniaturized lensless sensor imaging for cell and microorganism visualization at point‐of‐care testing is currently a focus of research and is becoming more necessary for cost‐effectiveness, portability, sensitivity and ease of use with limited resources and could be used to measure our developed red blood cell Golden Ratio (Fig. 1) (Gurkan et al. 2011). Due to the important microenvironment factors that drive the zeta potential and the DEP EMFFF within this cell, it is important to study and analyze this cell with as little disruption the microenvironment within where it resides. The authors suggest that once an erythrocyte is removed from the host, the quantum microenvironment that is a critical component of this field driven cell is disturbed and the Newtonian fluidics and calculations such as a Reynolds number may indeed be adversely altered. LBA and the future lensless imaging of a newly drawn drop of blood to obtain the red blood cell Golden Ratio could be valuable and accurate tools for evaluating the health and efficiency of the erythrocyte. (Wang and Popel 1993; Liao et al. 2013; Giovanna 2014).

## Conflict of Interest

None declared.

## Supporting information




**Video S1.** Red Blood Cells after BFA Treatment in Study Participant showing polarized movement and enhanced electrophoretic mobility.
**Video S2.** Red Blood Cells before BFA Treatment in Study Participant showing no apparent polarized movement with decreased electrophoretic mobility.
