# Erratum

**DOI:** 10.14814/phy2.13795

**Published:** 2019-07-19

In Purnell et al. (2018), the following errors were published in the article.

The Figure 4 supplied in the final version is incorrect and should instead be the following:

We apologize for the errors.

**Figure 4:**
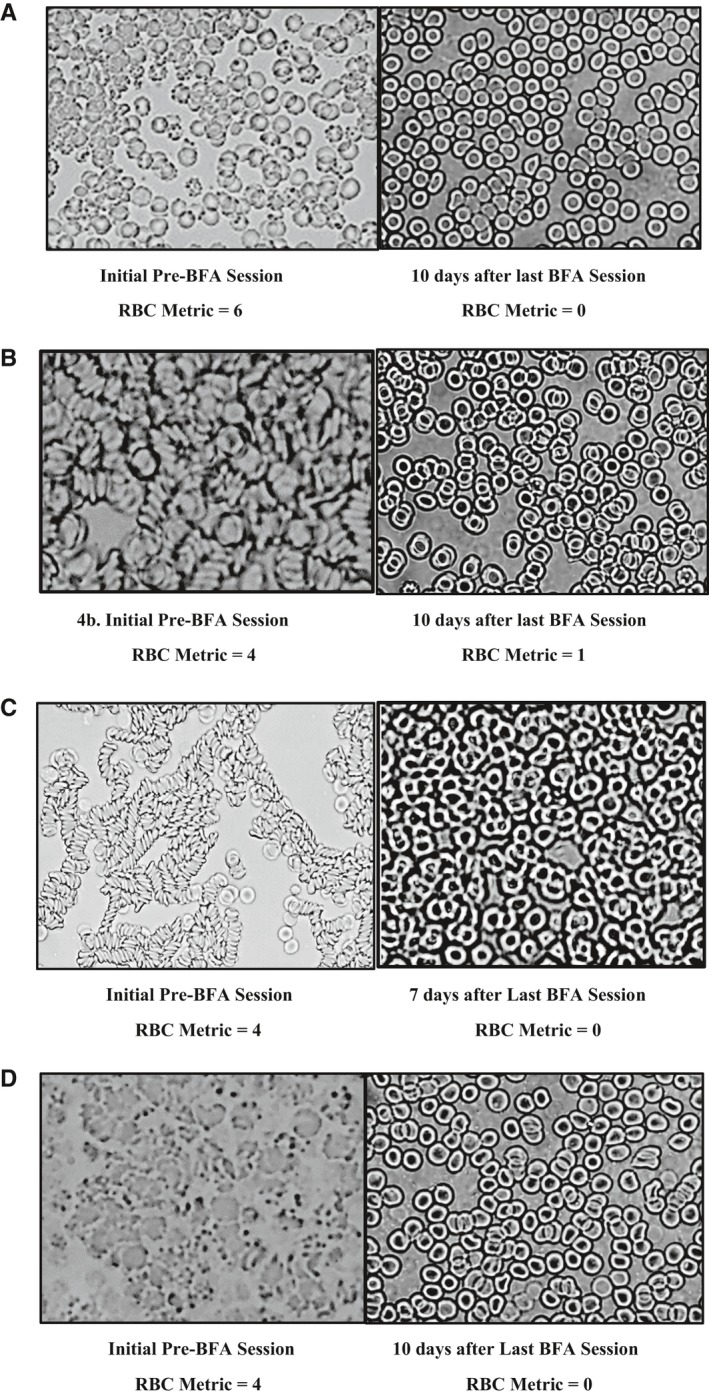
LBA changes (RBC Metric scores) noted from the Initial LBA taken prior to the first BFA Session in the study participants and the Final visit LBA which was taken 7‐10 days after the end of the 2 week study (After 6 BFA Immersion Therapy Session visits). The participants did not have a BFA session prior to the Final visit LBA.
