# Supplementary material for: Bio‐field array: a dielectrophoretic electromagnetic toroidal excitation to restore and maintain the golden ratio in human erythrocytes
Source: Physiol Rep. 2018 Jun 10;6(11):e13722. doi: 10.14814/phy2.13722 (PMC5995311; doi:10.14814/phy2.13722)
Supplement: Supplementary file 1 — Video S1. Red Blood Cells after BFA Treatment in Study Participant showing polarized movement and enhanced electrophoretic mobility. Video S2. Red Blood Cells before BFA Treatment in Study Participant showing no apparent polarized movement with decreased electrophoretic mobility. [file PHY2-6-e13722-s001.docx]

**Video 1. Red Blood Cells after BFA Treatment in Study Participant showing polarized movement and enhanced electrophoretic mobility.**

**Video 2. Red Blood Cells before BFA Treatment in Study Participant showing no apparent polarized movement with decreased electrophoretic mobility.**
